# The sequence and characterization of mitochondrial of *Lepus oiostolus* (Lagomorpha: Leporidae)

**DOI:** 10.1080/23802359.2020.1768930

**Published:** 2020-05-20

**Authors:** Xuze Zhang, Lin Fu, Songchang Guo

**Affiliations:** a College of Ecological Environment and Resources, Qinghai Nationalities University, Xining, China; b Qinghai Provincial Key Laboratory of Animal Ecological Genomics, Xining, China; c Center of life science, School of life sciences, Yunnan University, Kunming; d College of Animal Science and Technology, Hunan Agricultural University, Changsha, China

**Keywords:** *Lepus oiostolus*, mitochondrial genome, phylogenetic relationship

## Abstract

*Lepus oiostolus* is widely inhabited in the Qinghai-Tibet Plateau. So far, little mitochondrial genome information of this genus has been described. To grasp a better comprehension on the molecular basis of *L. oiostolus*, we obtained the complete mitochondrial DNA genome sequences of this species. The mitogenome was 17,320 bp in length, which consists of 13 protein-coding genes, 22 tRNA genes, 2 rRNA genes, and 1 noncoding regions. The complete mitochondrial genome of *L. oiostolus* would be of great utility in the phylogenetic analysis of the Lagomorpha and also provide meritorious insights into the deeper problems of the phylogenic analysis.

*Lepus oiostolus* is an endemic species in the Qinghai-Tibet Plateau, also known as gray-tailed hare. It belongs to the genus Lepus (Hodgson 1840; Kao and Feng 1964). In this study, the sample of *L. oiostolus* was collected from Fenxiakou, MenYuan Country, Qinghai Province, China (37.6160 N; 101.3132E). The genome DNA was extracted from the muscle tissue, using a modified method from the standard phenol/chloroform extraction process (Sambrook et al. 1989). The specimen of *L. oiostolus*, named as HB-01 was stored in the animal specimen room in Herbarium of College of Ecological Environment and Resources, Qinghai Nationalities University.

The complete mitochondrion genome of *L. oiostolus* was sequenced using the next-generation sequencing with Illumina Hiseq platform. The trimmed reads were mainly assembled by SPAdes (Bankevich et al. 2012). The whole sequence was annotated using the software Generous v11.1.5, and tRNA genes were predicted using online software MITOS (Bernt et al. 2013).

The mitochondrial genome of *L. oiostolus* was 17,320 bp in length, of which 16,124 nucleotides are coding DNA. This mitochondrial DNA sequence has been deposited in GenBank (accession NO. MT376741). It is a double-stranded closed loop structure composed of 13 protein-coding genes (PCGs), 2 rRNAs, 22 tRNAs and a D-Loop region. The overall base composition of the whole mitochondrial genome is 32% A, 29.4% T, 25.6% C, and 13.1% G, exhibiting obvious AT bias (61.4%).

Phylogenetic relationships of *L. oiostolus* with 15 species of *Lepus* from GenBank were resolved by means of Neighbor-joining (NJ) and an *Oryctolagus cuniculus* as outgroup. The NJ tree was built using MEGA 7 (Kumar et al. 2016) with bootstrap set to 1000. The phylogenetic tree suggested that *L. oiostolus*, *L. tolai, L. capensis, L. corsicanus, L. timidus, L. othus, L. arcticus, L. townsendii, L. tibetanus subsp. pamirensis*, and *L. sinensis* have a closer relationship compared with other species studied (Figure 1). Hares may have reticulate evolution and resulted in the rapid radiation and speciation of Lepus (Wu et al. 2005; Liu et al. 2011). This study can provide more basic data on conservation, genetics and phylogeny of *L. oiostolus* for the future research.

**Figure 1.**
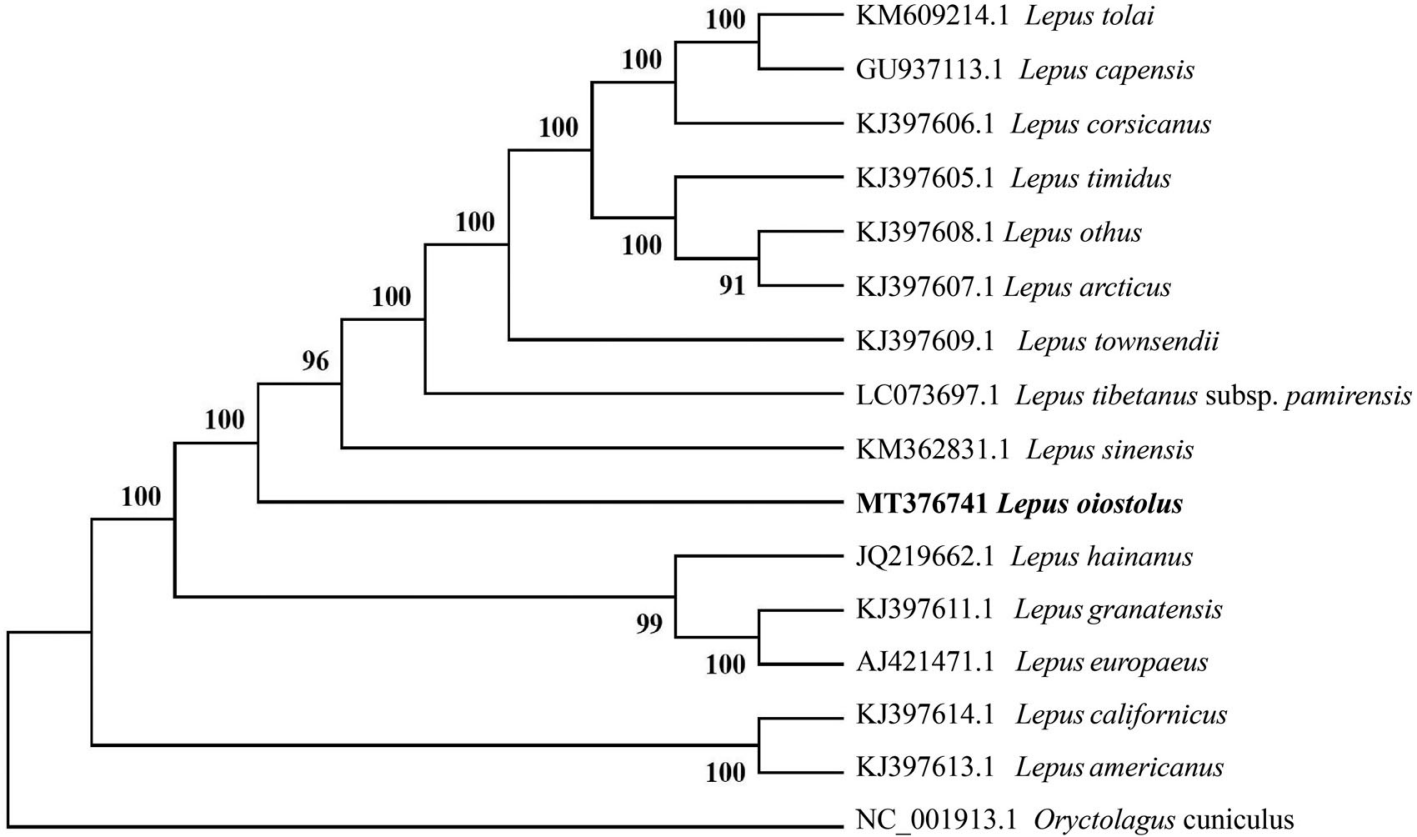
The Neighbor-joining tree based on 16 mitochondrial DNA sequences, an Oryctolagus cuniculus as outgroup.

## Data Availability

The data that support the findings of this study are openly available in Genbank at https://www.ncbi.nlm.nih.gov/genbank/, reference number MT376741.
